# Painkiller use in amateur football: high prevalence, but limited misuse

**DOI:** 10.1186/s13102-025-01396-9

**Published:** 2025-11-11

**Authors:** Andreas Kopf, Werner Krutsch, Dominik Szymski, Johannes Weber, Volker Alt, Hermann Josef Bail, Markus Geßlein, Johannes Ruether, Lorenz Huber

**Affiliations:** 1 Department of Orthopaedics and Traumatology, Paracelsus Medical University, Breslauer Straße 201, 90471 Nuremberg, Germany; 2 Department of Trauma Surgery, University Medical Centre Regensburg, Franz-Josef- Strauss Allee 11, 93053 Regensburg, Germany; 3 FIFA Medical Centre of Excellence, University Medical Centre Regensburg, 93053 Regensburg, Germany; 4 SportDocsFranken, 90473 Nuernberg, Germany

**Keywords:** Football, Soccer, Amateur sports, Medication, Painkiller use

## Abstract

**Background:**

The use of painkillers in football is a much-criticized topic, but there is hardly any scientific data at amateur level. The aim of the study is therefore to establish data on the prevalence of painkiller use in German amateur football. In addition, reasons for painkiller use and other influencing factors are investigated.

**Methods:**

In a cross-sectional analysis, the painkiller use among German amateur footballers was surveyed through an online protocol, involving players from the 4th league to the lowest amateur divisions.

**Results:**

Of the 604 participants, 489 were male (81.0%) and 115 were female (19.0%). The prevalence of painkiller use over the players’ entire careers in connection with football is approximately 77%. 75% of players report using painkillers only for acute injuries, with over two-thirds rarely or never taking them before a game. At 80%, female players report slightly higher painkiller use for acute injuries than male players (74.4%, *p* = 0.19). Age-related differences show that older players take painkillers more frequently than younger players (*p* < 0.001). Furthermore, league-specific differences show that higher playing levels (4th league) are associated with higher pain prevalence and higher painkiller use compared to the lowest five leagues (*p* < 0.001).

**Conclusions:**

This is the first study to record the use of painkillers in amateur football from the players’ perspective with a large sample size. The lifetime prevalence of painkiller use among amateur football players in this study matches levels found in previous studies of amateur and professional sports. Most players take painkillers for medically justified reasons, with misuse being minimal. However, a minority of players show less responsible usage. Further research into the contextual factors influencing painkiller use is needed, and educational initiatives are important to improve awareness regarding pain management in football.

**Supplementary Information:**

The online version contains supplementary material available at 10.1186/s13102-025-01396-9.

## Background

Painkiller use in football has gained significant attention across various media and political platforms in recent years [1, 2]. The relevance of this topic is underscored by the large number of active players in the German Football Association, with approximately seven million members, making football the most popular sport in Germany [3]. As regular sporting activity is also associated with pain and the risk of injury, proper pain and injury management is crucial to ensuring the health-promoting benefits of sports outweigh the negative consequences like the risk of adverse events or long-term consequences of ignoring injuries and pain [4]. Therefore, research on this subject is particularly important for amateur football, as its findings and educational outcomes could benefit a broad demographic. However, there is a significant lack of data in this area, as highlighted in a recent systematic review by Leyk et al. (2023) in the German Medical Journal, which calls for further research in this population [5]. Most previous studies on painkiller use in football focus almost exclusively on professional players, with conclusions for amateur athletes being extrapolated from studies in other sports. Data from various endurance events, such as the Hannover and Bonn marathons, indicate that the prevalence of painkiller use among participants ranges from 17 to over 60% [1, 6].

Studies commissioned by the FIFA for the World Cups from 2002 to 2014, which investigated the use of medication at the tournaments by asking the team medical staff, are an essential part of the football-specific data situation. In summary, these show that the use of painkillers in football, especially in professional football, has remained at a consistently high level over the years, with prevalence of over 50% throughout the course of the World Cup tournaments [7–10]. Further studies show that the use of painkillers is slightly lower in professional youth football [11]. Non-steroidal anti-inflammatory drugs are the most commonly used group of painkillers in football and all other sports. However, almost all studies were conducted at major tournaments such as the FIFA World Cup, and only a few in professional football leagues. The special conditions of these tournaments, such as the big social pressure, media attention and the high intensity of stress over a very limited duration of the competitions, could favour an increased use of pain medication [12].

Data from a few studies examining painkiller use over an entire season in various professional leagues indicate even higher prevalence rates in some individual cases [13–15]. The results of a meta-analysis with over 40,000 analysed data sets from youth sports found an increased point prevalence of painkiller use correlating with the level of performance, while the sensitivity analysis of unspecified analgesic use in the same study showed results the other way around, which at least raises doubts on the transferability of the data from the professional to the amateur sector [16].

In summary, there is almost no data in the literature on painkiller use specifically in amateur football, highlighting a significant research gap. Moreover, the transferability of data from professional to amateur football remains unclear and appears rather unlikely.

Accordingly, this study project aims to assess the current state of painkiller use in German amateur football. The primary objective is to illustrate the prevalence of painkiller use among German amateur football players. Secondary objectives include analysing the indications and reasons for painkiller use, as well as identifying influencing factors.

## Methods

This cross-sectional study was conducted using an online protocol targeting players in German amateur football from the fourth league to the lowest amateur divisions. Both male and female players were included. Data collection occurred between January and July 2022. All analysed football teams belong to the regional football Association in Germany, the Bavarian Football Association (BFV), with more than 1.6 million football players and more than 4500 football clubs. The study group and the BFV have a reliable partnership for over 10 years to reach out to amateur footballers. Initial contact via email and telephone was made with the responsible officials at the clubs, who then passed the study information on to the coaches and players. Further communication regarding the informed consent process and the distribution of questionnaires was conducted with the players themselves.

In that way, 54 Clubs, equally distributed through the investigated divisions, were reached. All participants were thoroughly informed about the study methodology and the online protocol and were encouraged to complete it truthfully. Players actively competing in amateur football during the 2021/2022 season who completed the questionnaire in full were included, while incomplete responses were excluded. There were no further in- or exclusion criteria regarding for example social desirability.

Pain was defined according to the terminology of the International Association for the Study of Pain (IASP) as an unpleasant sensory and emotional experience associated with actual or potential tissue damage, or described in terms of such damage [17].

The online protocol consisted of 46 standardized items. These were based on the consensus statements for scientific studies in football [18], previous studies on the use of nutritional supplements and painkillers in football [8, 12] and the most common types of injury in football [19]. Key objectives included injury history, frequency of sports-related pain, medication types and dosages, and players’ perspectives on painkiller use. Particular attention was also paid to painkiller access in locker rooms and attitudes toward their compatibility with football. Misuse was defined as the use of painkillers for prevention, performance enhancement, maintaining performance despite injuries that require rest and recovery, or against medical advice. This protocol was also used in a previously published study that assessed the use of painkillers in amateur football from the coaches’ perspective [20]. After the questions were compiled, they were reviewed by experts from the medical commission of the German and the Bavarian football association.

IBM SPSS Statistics Version 28.0 (IBM SPSS Inc, Chicago, IL, USA) was used for the statistical analysis and graphical presentation of the tables and figures. Descriptive statistics were calculated for all variables, with continuous variables expressed as the mean and standard deviation (SD). For subgroup comparison Chi- square tests were obtained and the confidence interval (CI) was set to 95%.

This study was approved by the Ethics Committee of the University of Regensburg (No. 22–2781-101).

## Results

A total of 863 amateur football players accepted the invitation to participate in the study. Of these, 604 (70.0%) fully completed the protocol and were therefore included in the analysis, comprising 489 male (81.0%) and 115 female players (19.0%, Table 1). The majority of the surveyed players compete in district and state leagues, with the former highest divisions being, on average, classified at the same level (Table 2). Lastly, only 4.8% (*n* = 29) of the players work in the medical field.

**Table 1:** Anthropometric and football-specific data of the study population

	Mean ± SD
Age in years	24.9 **±** 5.3
Height in cm	178.9 **±** 8.5
Weight in kg	76.4 **±** 12.9
Sex = male, *n (%)*	489 (81.0%)
Medical profession	29 (4.8%)
Paid amateur football, *n (%)*	215 (35.6%)
Unpaid amateur football, *n (%)*	389 (64.4%)

**Table 2:** Current and former highest league

German leagues	Current league n (%)	Former highest league n (%)
Bundesliga		11 (1.8%)
2nd Bundesliga		9 (1.5%)
3rd Bundesliga		8 (1.3%)
4th division	50 (8.3%)	63 (10.4%)
5th division	34 (5.6%)	95 (15.7%)
6th division	131 (21.7%)	138 (22.8%)
7th division	137 (22.7%)	146 (24.2%)
8th division	58 (9.6%)	69 (11.4%)
9th division	77 (12.7%)	41 (6.8%)
10th division	70 (11.6%)	17 (2.8%)
11th division	29 (4.8%)	7 (1.2%)
12th division	18 (3.0%)	0 (0.0%)

Playing football is associated with pain due to strain and injury, 40% of the surveyed players experience football-related pain more than once a week. Over 80% report experiencing pain at least once a month, while less than 5% state that they never have football-related pain. The majority of players report various previous injuries, with only 5 participants (0.8%) indicating that they had never suffered any injuries. The most commonly reported injury types are contusions, muscle strains, and capsule/ligament injuries (Table 3).

**Table 3:** Frequency of football-associated pain and injuries

**Football-associated pain,** ***n*** **(%)**	
Every match and training	44 (7.3%)
Once a week	195 (32.3%)
Once a month	244 (40.5%)
Once every six month	98 (16.3%)
Not at all	22 (3.6%)
**Football-associated painkiller use in their career,** ***n*** **(%)**	
Already used once	465 (77.2%)
Never used	134 (22.8%)
**Football associated injuries during their career,** ***n*** **(%)**	
Contusion	442 (73.4%)
Ligament injury	379 (63.0%)
Muscle strain	355 (55.6%)
Skin injury (abrasion/laceration)	354 (58.8%)
Muscle fiber tear	211 (35.0%)
Fractures	193 (32.1%)
Back pain	297 (49.3%)
Cartilage/meniscus injury	117 (19.4%)
Capsule injury	230 (38.2%)
Groin/hip pain	270 (44.9%)
Foot/heel pain	267 (44.4%)
Tendon pain (patella/achilles tendon)	152 (25.2%)
Disc/nerve pain	48 (8.0%)
Muscle bundle rupture	37 (6.1%)
Concussion	109 (18.1%)
Other	88 (14.6%)
None	5 (0.8%)

77.2% of all players state that they had taken painkillers at some point for injuries and complaints directly related to playing football (Table 4). Additionally, almost 80% of players have already been prescribed pain medication by a doctor. More than half of all respondents have also bought painkillers over the counter without a doctor’s advice. Additionally, regarding the sources of supply of painkillers, more than 60% state that they obtain their pain medication themselves. All other sources of supply represent a significantly lower proportion. Only a quarter of players (24.3%) obtain their painkillers exclusively from medical professionals. Furthermore, leftover medication as a result of medical treatment was reported by approximately two-thirds (65.0%) of amateur players (Table 4).

**Table 4:** Painkiller use and availability for amateur football players

**Medically prescribed painkillers,** ***n*** **(%)**	
Ever received	469 (77.9%)
Never received	133 (22.1%)
**Painkillers purchased over-the-counter,** ***n*** **(%)**	
Ever acquired	351 (58.4%)
Never acquired	250 (41.6%)
Trainers	8 (1.3%)
Team physician/supervising physician	145 (24.3%)
Physiotherapist	78 (13.1%)
Teammates	69 (11.6%)
Family	212 (35.6%)
Purchased by themselves	365 (61.2%)
None of the above	52 (8.7%)
**Painkillers left over after medical treatment,** ***n*** **(%)**	
Always	197 (30.1%)
Often	208 (35.0%)
Occasionally	78 (13.1%)
Rare	54 (9.1%)
Never	76 (12.8%)

The majority of pain medication is taken for acute injuries and complaints, as stated by around 75% of all respondents. Chronic complaints, on the other hand, are a reason for using pain medication in only 12.5% of cases. Two-thirds have never or rarely taken painkillers before a game. However, a minority of amateur players (10.6%) frequently use painkillers during competition. Similarly, around 10% of players consider painkillers and competition to be completely or highly compatible, whereas half of the amateur players (50.5%) regard the use of painkillers in competition as barely or not at all compatible. Finally, only a small minority (3.2%) report prophylactic painkiller use often or always, while the majority (90.2%) do so rarely or never (Table 5).

**Table 5:** Reasons for painkiller use, compatibility of painkillers and football and prophylactic use

**Reasons to take painkillers,** ***n*** **(%)**	
Acute injuries	452 (75.5%)
Chronic injuries	75 (12.5%)
When prescribed by a doctor	231 (38.6%)
Decided by themselves	190 (31.7%)
None of the above	21 (3.5%)
**Ever used of painkillers before matches,** ***n*** **(%)**	
Always	5 (0.8%)
Often	59 (9.8%)
Occasionally	136 (22.6%)
Rare	205 (34.1%)
Never	196 (32.6%)
**Compatibility of painkillers and competition,** ***n*** **(%)**	
Fully compatible	18 (3.0%)
Very compatible	44 (7.3%)
Predominantly compatible	235 (39.2%)
Predominantly incompatible	226 (37.7%)
Not compatible	77 (12.8%)
**Prophylactic use of painkillers,** ***n*** **(%)**	
Always	3 (0.5%)
Often	16 (2.7%)
Occasionally	40 (6.7%)
Rare	83 (13.8%)
Never	459 (76.4%)

Regarding differences in the subgroups, no statistically significant difference was found between male and female players. However, female football players report slightly higher use of painkillers for acute injuries compared to their male counterparts (80.0% vs. 74.4%, *p* = 0.19) and are more likely to follow doctors’ prescriptions (44.3% vs. 37.2%, *p* = 0.16).

In terms of age groups, 28.0% of players under the age of 20 report experiencing football-related pain at least once a week, whereas players over 30 report it more frequently, with 47.5% experiencing pain at least once a week. Alongside the frequency of football-related pain, the proportion of players over 30 who had taken painkillers for football-related pain, is significantly higher than that of players under 20 (89.1% vs. 63.4%, *p* < 0.001).

Furthermore, the frequency of football-related pain was found to be significantly higher among players in the highest amateur division, the fourth league, than among players in the lower divisions. Compared to leagues from the 5th to 9th divisions, which represent performance-oriented amateur football, the 4th league shows a higher prevalence of players experiencing pain more than once a week (46.0% vs. 29.6%, *p* < 0.001).

Corresponding to the higher prevalence of pain in the upper divisions of amateur football, a larger proportion of players in the 4th league (84.0%) has already taken painkillers due to football-related pain. In comparison, only 71.9% of players in the lowest 5 amateur leagues report having taken painkillers for football-related pain (*p* < 0.001, Table 6).

**Table 6:** Differences in pain and painkiller use between sexes, age groups and divisions

**Reasons to take painkillers,** ***n*** **(%)**	**Men**	**Women**	***p*** **-value**
Acute injuries	360 (74.4%)	92 (80.0%)	0.19
Chronic injuries	60 (12.4%)	15 (13.0%)	0.94
When prescribed by a doctor	180 (37.2%)	51 (44.3%)	0.16
Decided by themselves	150 (31.0%)	40 (34.8%)	0.46
None of the above	19 (3.9%)	2 (1.7%)	0.40
**Football-related pain,** ***n*** **(%)**	**Age under 20**	**Age over 30**	**p-value**
Every match and training	3 (3.7%)	12 (11.9%)	0.08
Once a week	20 (24.4%)	36 (35.6%)	0.14
Once a month	35 (42.7%)	32 (31.7%)	0.17
Once every six months	18 (22.0%)	18 (17.8%)	0.61
Not at all	6 (7.3%)	3 (3.0%)	0.31
**Football-related painkiller use,** ***n*** **(%)**	**Age under 20**	**Age over 30**	**p-value**
Already used once	52 (63.4%)	90 (89.1%)	**<0.001
Never used	30 (36.6%)	11 (10.9%)	**<0.001
**Football-related pain,** ***n*** **(%)**	**4th league**	**5th −9th league**	**p-value**
Every match and training	3 (6.0%)	17 (5.6%)	*0.040
Once a week	23 (46.0%)	89 (29.6%)	**<0.001
Once a month	15 (30.0%)	132 (43.9%)	**<0.001
Once every six months	5 (10.0%)	53 (17.6%)	**<0.001
Not at all	4 (8.0%)	10 (3.3%)	0.06
**Football-related painkiller use,** ***n*** **(%)**	**Forth league**	**Lowest 5 leagues**	**p-value**
Already used once	42 (84.0%)	174 (71.9%)	**<0.001
Never used	8 (16.0%)	68 (28.1%)	**<0.001

*p < 0.05 **p < 0.01

## Discussion

### Prevalence and reasons for painkiller use

This study provides new insights into the prevalence of painkiller use in amateur football from the players’ perspective—an area identified as a gap in the scientific literature, including the review by Leyk et al. in the German Medical Journal [5]. 77.2% of the players in this study have already used painkillers because of football-related pain. 75% of the study population report taking painkillers for acute injuries, while 10% use them for chronic issues. This suggests that most athletes have a medical reason for their use. The most common injuries cited are bruises and ligament injuries.

Previous studies involving professional football players have reported comparable painkiller use, averaging approximately 70%, with prevalence varying depending on whether a single season or the entire playing career is considered [5, 12].

Some longer studies covering an entire season report even higher prevalence, reaching up to 90% [14, 15]. However, most research focuses on short-term periods, such as World Cup tournaments [9, 10, 12], so that direct comparability is difficult. In contrast, studies from other sports suggest widespread painkiller use over an entire season. For example, up to 95% of college American football players take painkillers at least once per season [20]. A previously published preliminary study assessed team coaches’ perspective on the prevalence of painkiller use in German amateur football. The data published in that study show significantly lower values, with coaches reporting that they assume approximately 36% of their players have taken painkillers at least once due to football-related pain [21].

However, this study found that 67% of amateur footballers avoid painkillers before matches. Additionally, over 90% oppose using painkillers prophylactically, marking the first time this has been studied in amateur football. Other studies report much higher prophylactic use in different sports, such as the Bonn Marathon, where 61% of runners took NSAIDs before the race [1, 22]. On the other hand, a study on youth basketball found similarly low prophylactic use (5%) [23].

## Access and availability of painkillers

Besides use frequency and reasons, this study also examines factors influencing painkiller behavior. Previous research suggests that coaching styles can impact players’ willingness to compete while injured [24], but only 1.3% of players in this study received painkillers directly from their coaches. The most common sources are personal over-the-counter purchases (60%) and team doctors. Notably, many players (about two-thirds) reported leftover prescribed painkillers, raising concerns about easy access leading to overuse. These findings highlight two key areas for future education: (1) Players should receive direct education on responsible painkiller use and (2) medical staff should be more aware of leftover medications that might contribute to excessive use.

## Differences between sexes, age and level of play

The sex distribution of all players in the BFV is 85% men and 15% women, the study cohort reflects this distribution with 489 male (81.0%) and 115 female participants (19.0%) [25]. When it comes to the use of painkillers for acute injuries, female players are more likely to use painkillers (80%) than males (70%). This increased use of painkillers by female players was also demonstrated by Hager et al. (2021) in amateur volleyball players. In their study, 60% of female players use over-the-counter painkillers, compared to only 38% of male volleyball players [26]. This may relate to sex differences in pain perception, as postulated by Bartley et al. (2013), even if more precise etiological correlations are still unclear [27].

There are also differences in the frequency of pain and painkiller use within age groups. Players under 20 years report less pain during training than those over 30. Older players have more experience with painkillers (90%) compared to younger players (63%), a pattern also observed in professional football [11, 12]. The results of this study show that the use of painkillers by amateur football players significantly increases with age, which of course correlates with the significantly increased prevalence of football-related pain with increasing age. These findings are comparable with the results from professional football in other studies [5, 11, 12].

Further significant differences are found across competition levels, consistent with previous studies [5, 16]. Players in the 4th league (the highest amateur level) report more weekly pain (52%) than mid-level amateurs (35%). Similarly, 84% of 4th league players have taken painkillers for football-related reasons, compared to 72% in lower divisions. This aligns with findings from youth sports, where higher-performing athletes use painkillers more frequently [16]. Similar trends have been observed in professional tournaments, where painkiller use increases in the knockout stages [10].

### Study limitations and strengths

Due to the design of an online questionnaire-based cross-sectional study, there are some limitations, particularly the possibility that responses were influenced by social desirability.

Furthermore, the recruitment of the participating teams was limited to the federal state of Bavaria, as the extensive network in Bavarian amateur football and the associated target group-specific approach were intended to enhance participant compliance, which does not completely rule out a possible selection bias. However, no methodological weaknesses were identified that would prevent the transferability of the results to the entirety of German amateur football. This is because the Bavarian regional association has its own 4th league, which independently represents the highest amateur division in football.

As the studied population varies significantly in some cases across subgroups such as age, gender, and league affiliation, comparability between these subgroups may be limited. However, the overall population of this study includes a very large number of participants, which strengthens its general validity. Additionally, the inclusion of senior men’s teams from the lowest amateur divisions, with a considerable age difference, may further limit comparability, as older players in particular tend to have a distinct pattern of painkiller use—a finding that this study was able to confirm once again.

A particular strength of this study is the fact that most of the data was collected for the first time, as amateur football has often been neglected as a research focus. While this limits direct comparability with previous studies, it also provides a valuable foundation for future research and offers important insights and starting points for educational initiatives. The study’s large population of over 600 players is especially noteworthy given the general lack of data in amateur sports and the typically low response rates in this area.

## Conclusions

This study presents, for the first time, the current status of painkiller use in German amateur football, revealing a prevalence comparable to values found in studies on professional football. Amateur football players primarily take painkillers for medical reasons, and the majority reject their prophylactic use in football. Educational initiatives should be implemented to inform the small number of players who take painkillers prophylactically. This could be achieved either through direct education or by providing more detailed information to coaches and medical staff, as both groups play an important role in influencing painkiller use in football. In addition, it must also be informed that the use of painkillers may result in potential anti-doping rule violations.

A key focus of these educational efforts should be on explaining the indications for painkillers, their potential side effects, and the reasons they should not be taken prophylactically in football. While further scientific research on painkiller use in football is necessary, educational initiatives based on the findings of this study can already be implemented, particularly in the amateur football setting. Since most studies on painkiller use in sports have been conducted in football, similar educational programs aimed at preventing painkiller misuse should also be introduced and carried out in other sports.

## Supplementary Information

Supplementary Material 1.

## Data Availability

Data and materials can be sent on reasonable request to the corresponding author ([lorenz1.huber@ukr.de](mailto: lorenz1.huber@ukr.de)). The protocols, used in this study, are supplemental material to this paper.
