# Supplementary material for: Painkiller use in amateur football: high prevalence, but limited misuse
Source: BMC Sports Sci Med Rehabil. 2025 Nov 11;17:325. doi: 10.1186/s13102-025-01396-9 (PMC12604260; doi:10.1186/s13102-025-01396-9)
Supplement: Supplementary file 1 — Supplementary Material 1. [file 13102_2025_1396_MOESM1_ESM.pdf]

Universitätsklinikum Regensburg

Athlete survey on the use of painkillers in amateur football

1. Age	Click or tap to enter text.
2. Gender	☐ M ☐ W
3. Size	Click or tap to enter text.
4. Weight	Click or tap to enter text.
5. Do you work in a medical profession?	☐ Yes ☐ No
6. Currently in which division?	Click or tap to enter text.
7. How many days a week do you play football?	Click or tap to enter text.
8. What was your top division?	Click or tap to enter text.

9. How often do you have non-exercise pain, such as headaches or back pain?

☐ Always ☐ Often ☐ Occasionally ☐ Seldom ☐ Never

10. How often do you have pain in connection with your sport?

☐ Every matchday & training ☐ 1 time a week ☐ 1 time per month
☐ 1x per half a year ☐ Not at all

11. How much do you differentiate in your perception of pain between positive (e.g. sore muscles after hard training) and negative (e.g. overload or injury) pain?

☐ Full ☐ Strong ☐ Mediocre ☐ Weak ☐ Not at all

12. How much do you differentiate between self-inflicted and third-party pain in your pain perception and treatment? (In the sense of: "I tend to be more careful with pain after an intense duel")

☐ Full ☐ Strong ☐ Mediocre ☐ Weak ☐ Not at all

13. How much do you differentiate between pain perception and treatment before and after the competition? (In the sense of: "Before the competition, I don't perceive pain as such")

☐ Full ☐ Strong ☐ Mediocre ☐ Weak ☐ Not at all

14. Former own injuries or complaints (multiple answers possible):

☐ Bruises ☐ Capsular injuries ☐ Ligament injuries

☐ Pulled muscle ☐ Hamstring ☐ Muscle bundle tear

☐ Concussion ☐ Broken bones ☐ Cartilage/Meniscus injuries

☐ Skin injuries(abrasion/laceration) ☐ Back pain

☐ Discs-/Neuralgia ☐ Groin/hip pain ☐ Tendon pain

☐ Foot/heel pain ☐ Other ☐ None

15. Have you ever received a prescription for painkillers from your doctor?

☐ Yes ☐ No

16. Have you ever bought pain medication at the pharmacy without a prescription?

☐ Yes ☐ No

Question for yes: What painkillers did you buy?

☐ Ibuprofen ☐ Diclofenac ☐ Novalgin ☐ Arcoxia

☐ Paracetamol ☐ Aspirin ☐ Opioids (Tramal/Oxycodone/Tilidine, etc.)

☐ CBD (e.g. CBD oil) ☐ Other (free text) ☐ None

17. What painkillers have you taken yourself? (Multiple answers possible)

☐ Ibuprofen ☐ Diclofenac ☐ Novalgin ☐ Arcoxia

☐ Paracetamol ☐ Aspirin ☐ Opioids (Tramal/Oxycodone/Tilidine, etc.)

☐ CBD (e.g. CBD oil) ☐ Other (free text) ☐ None

18. What form of painkillers did you receive? (Multiple answers possible)

☐ Tablets/Capsules ☐ Squirt ☐ Infusions ☐ Suppository

☐ Anoint ☐ Powder ☐ None

19. How often do you take two or more painkillers at the same time?

☐ Always ☐ Often ☐ Occasionally ☐ Seldom ☐ Never

20. When do you take painkillers? (Multiple answers possible)

☐ In case of acute injuries/complaints ☐ For chronic injuries/complaints

☐ If it is prescribed ☐ Independently ☐ No information

21. Have you ever taken pain medication related to football?

☐ Yes ☐ No

If Yes:

For which of the following pains do you use painkillers the most? (Multiple answers possible)

☐ Positive pain ☐ Negative pain ☐ Pain without enemy impact

☐ Third-party fault ☐ Before the competition ☐ After the Pain Contest

☐ None of the above

On what occasion did you take it? (Multiple answers possible)

☐ Only after injuries ☐ Preventive action before the game

☐ Preventive before training ☐ General in everyday life

☐ None of the above

How often have you experienced pain after normal stressful training (sore muscles, etc.)
Need painkillers?

☐ Always ☐ Often ☐ Occasionally ☐ Seldom ☐ Never

22. Which type of injury or discomfort has ever been the reason for taking it? (Multiple answers possible)

☐ Bruises ☐ Capsular injuries ☐ Ligament injuries ☐ Pulled muscle

☐ Hamstring ☐ Muscle bundle tear ☐ Concussion ☐ Broken bones

☐ Cartilage/Meniscus injuries ☐ Skin injuries (abrasion/laceration)

☐ Back pain ☐ Discs-/ Neuralgia ☐ Groin/hip pain ☐ Tendon pain ☐ Foot/heel

☐ Other ☐ None

23. How often were the painkillers prescribed by the doctor?

☐ Always ☐ Frequently ☐ Occasionally ☐ Seldom ☐ Never

24. Who do you get your painkillers from? (Multiple answers possible)

☐ Coach ☐ Team doctor/supervising physician ☐ Physiotherapist

☐ Teammates ☐ Family ☐ Self-purchased ☐ None of the above

25. How often have you taken painkillers before training for sports injuries that are still painful?

☐ Always ☐ Often ☐ Occasionally ☐ Seldom ☐ Never

26. How often did you take painkillers before the game because of sports injuries that were still painful?

☐ Always ☐ Often ☐ Occasionally ☐ Seldom ☐ Never

27. How much do you agree that there are phases in a season when you use pain medication more often?

☐ Full ☐ Strong ☐ Mediocre ☐ Weak ☐ Not at all

28. To what extent are typical football-typical phases, such as an important match, a season or cup final, reasons for taking painkillers?

☐ Full ☐ Strong ☐ Mediocre ☐ Weak ☐ Not at all

29. Which of the following factors is another reason for taking painkillers? (Multiple answers possible)

☐ Competition intensity ☐ Pressure ☐ Family/professional pressure
☐ Free text ☐ None of these reasons

30. How often do you take pain ointments preventively before or after the game?

☐ Always ☐ Often ☐ Occasionally ☐ Seldom ☐ Never

31. How often have you taken pain medication as part of the medical treatment for the return to sport?

☐ Always ☐ Often ☐ Occasionally ☐ Seldom ☐ Never

32. How often is there anything left over from prescribed painkillers after treatment has ended?

☐ Always ☐ Often ☐ Occasionally ☐ Seldom ☐ Never

33. How often do you take pain medication so that you don't have a drop in performance with minor injuries?

☐ Always ☐ Often ☐ Occasionally ☐ Seldom ☐ Never

34. How much do you agree that painkillers increase your performance?

☐ Full ☐ Strong ☐ Mediocre ☐ Weak ☐ Not at all

35. How often do you take pain medication prophylactically or routinely because it allows you to play harder or more fearlessly in the game?

☐ Always ☐ Often ☐ Occasionally ☐ Seldom ☐ Never

36. To what extent do you see the use of pain medication as compatible with competition?

☐ Fully compatible ☐ Very compatible ☐ Mostly compatible
☐ Mostly incompatible ☐ Incompatible

37. To what extent do you see the use of pain medication as compatible with training?

☐ Fully compatible ☐ Very compatible ☐ Mostly compatible
☐ Mostly incompatible ☐ Incompatible

38. In your opinion, should certain painkillers continue to be available over the counter?

☐ Yes ☐ No

39. In your opinion, should common painkillers be on the doping list in competitive sports?

☐ Yes ☐ No

40. How worrying do you think the side effects of pain medication are?

☐ Full ☐ Strong ☐ Mediocre ☐ Weak ☐ Not at all

41. What side effects of classic pain medications, such as ibuprofen or diclofenac, are you aware of? (Multiple answers possible)

☐ Stomach damage ☐ Kidney damage ☐ Cardiovascular damage

☐ Liver damage ☐ Other ☐ None

42. Are you aware of any other effects of common painkillers?

Click or tap to enter text.

43. Have you ever found yourself in a situation where you felt pressured by one of the following players to play despite pain or using painkillers? (Multiple answers possible)

☐ Coach ☐ Team doctor/supervising physician ☐ Physiotherapist

☐ Teammates ☐ Family ☐ Own claim ☐ Never

☐ None of the above

44. How detailed do you feel you are informed about the topic of painkiller abuse in sport?

☐ Full ☐ Strong ☐ Mediocre ☐ Weak ☐ Not at all

45. Have you received information material on the abuse of painkillers?

☐ Yes ☐ No

46. When do you perceive the use of painkillers as abuse? (Multiple answers possible)

☐ Always ☐ Never

☐ Taking before the game because of pain ☐ Taking before training for pain

☐ Prophylactic use before the game ☐ Prophylactic use before training

☐ None of the above
